# Involuntary Hospitalization of People Living with Dementia: A Comparative Policy Analysis

**DOI:** 10.1093/geroni/igaf122.2191

**Published:** 2025-12-31

**Authors:** Clark Benson, Lea Gardiner, Laura Block, Andrea Gilmore-Bykovskyi

**Affiliations:** University of Wisconsin–Madison School of Medicine and Public Health, Madison, Wisconsin, United States; University of Wisconsin–Madison, Madison, Wisconsin, United States; University of Wisconsin-Madison, Madison, Wisconsin, United States; University of Wisconsin-Madison School of Medicine and Public Health, Madison, Wisconsin, United States

## Abstract

Neuropsychiatric symptoms, such as agitation and aggression, affect virtually all individuals with dementia and can sometimes be challenging to manage. These symptoms can result in crises characterized by psychological or physical threat to self and others. In some cases, these crises lead to involuntary hospitalization, a controversial policy typically used for people living with mental illness that involves the person being hospitalized and treated without their consent. Involuntary hospitalization policies vary by state, requiring local approaches to understanding their impact on people living with dementia. Rates of involuntary hospitalization among people with dementia and evaluation of existing policies for this practice are largely unknown. As a first step, this study conducts a comparative analysis of Wisconsin and Idaho state policies to lay the groundwork for subsequent research on their application. Wisconsin has required use of an “Emergency Protective Placement” policy since 2012 as involuntary hospitalization was declared unconstitutional for people living with dementia. In contrast, Idaho created and implemented a “Crisis Hold Policy” in 2024 to provide acute crisis stabilization services for people living with neurological conditions, including dementia. Comparative analysis reveals both state policies allow people living with neurological conditions to be hospitalized for a limited amount of time. While Wisconsin’s policy has strict facility and treatment restrictions, Idaho’s policy is more flexible and systematically collects data. This analysis generates three dementia crisis policy evaluation domains for consideration in future research and evaluation, including access to acute stabilization facilities, barriers to acute treatment, and data provision and government accountability.

